# Downregulation of miR‐1225‐5p is pivotal for proliferation, invasion, and migration of HCC cells through NFκB regulation

**DOI:** 10.1002/jcla.23474

**Published:** 2020-07-28

**Authors:** Lin Liu, Weiguo Zhang, Yujing Hu, Liangliang Ma, Xiangsu Xu

**Affiliations:** ^1^ Department of Oncology Hematology People's Hospital of Linzi District Zibo China; ^2^ Department of General Surgery Tianjin Fifth Central Hospital Tianjin China; ^3^ Department of Obstetric Area 3 Shandong Qilu Hospital Pingyi Branch (Pingyi County People's Hospital) Linyi China; ^4^ Department of Hepatolibiary Surgery Jining No.1 People's Hospital Jining China

**Keywords:** hepatocellular carcinoma, invasion, migration, miR‐1225, NFκB p65, proliferation

## Abstract

**Background:**

As one of the most frequently seen malignancies, hepatocellular carcinoma (HCC) serves as the second largest contributor to malignancy‐specific mortality worldwide. MicroRNA‐1225‐5p (miR‐1225) exerts an essential impact on the growth and metastasis of many malignancies. However, the contribution of miR‐125 to HCC and the molecular mechanism of cancer cell viability and apoptosis are still unclear. We focused our research on exploring the function and molecular mechanism of miR‐1225 in regulating HCC cell growth, migration, and invasion.

**Material:**

Quantitative PCR data showed that miR‐1225 expression was repressed in HCC cell lines and in the tissues of HCC patients, compared to that in normal human hepatic cells and tissues. Transfection of a miR‐1225 mimic inhibited cell viability and proliferation as indicated by CCK‐8 staining and MTT assay. Transwell invasion, wound healing assay, and Western blotting were performed to assess whether miR‐1225 repressed the metastasis and invasion of HCC cells, and decreased matrix metalloproteinase 9 (MMP9) expression. Further bioinformatic prediction and dual‐luciferase reporter assay suggested that miR‐1225 targeted the 3′‐UTR of NFκB p65.

**Results:**

Overexpression of p65 protein counteracted the repressive impact of miR‐1225 on invasion, migration, and proliferation of HCC cells.

**Conclusion:**

This research provided new evidences that miR‐1225 inhibits the viability, migration, and invasion of HCC cells by downregulation of p65.

## Graphical abstract

NFκB p65 is involved in miR‐1225‐inhibited proliferation, invasion, and migration of HepG2 and Huh‐7 cells. (A, B) HepG2 and Huh‐7 cells underwent co‐transfection with of pCDNA3‐p65/pCDNA3 and the miR‐1225 mimic to upregulate the p65 expression. (A, C) qPCR and (B, D) WB were performed to detect the p65 protein expression in both HCC cell lines. (E, F) Proliferation of Huh‐7 and HepG2 cells was measured at 12, 24, 36, 48, 60, and 72 h after transfection, using the MTT assay. (G, H) Invasion and (I, J) migration of HepG2 and Huh‐7 cells was examined using the Transwell migration test and wound healing assay. Outcome is displayed as mean ± SD. *P < .05 vs control group.

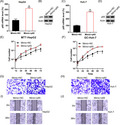

## 1 INTRODUCTION

Hepatocellular carcinoma (HCC) serves as one of the ten most prevalent malignancies worldwide with respect to incidence and mortality.^1^ It is characterized to have a bad clinical outcome and shows metastasis to distant regions.^2^, ^3^ Despite emerging studies investigating migration and invasion, metastasis is still the dominant contributor to HCC‐specific mortality as in other malignancies.^4^, ^5^, ^6^, ^7^, ^8^ Even though its diagnostic process and treatment have advanced substantially, its mortality rate has remained considerably high in the last 5 years. Therefore, there is an imperative need to investigate and understand the innate mechanism that dictates metastatic behavior in HCC cells.

According to clinical records, the high invasive capability of malignant cells is related to poor survival, suggesting the aggravating contribution of strong invasive capability to the malignant development of cancer.^9^, ^10^ In HCC, macrophagocytes observed inside primary neoplasms are a general indicator of tumor progression and metastasis.^11^, ^12^, ^13^, ^14^ Macrophagocytes, after activation, are critical for the development and invasive process of tumors as they selectively upregulate matrix‐metalloproteinases (MMPs), which disrupt the extracellular matrix and undermine the basement membrane.^15^

As small single‐stranded RNAs without coding functions, miRs contain eighteen to twenty‐two nucleotides and are endogenously expressed in multiple organisms ranging from animals to plants.^16^, ^17^ Various studies have revealed the essential impact of miRs on different biological reactions including apoptosis and migration in malignancy. Few studies have revealed the tumor repressive impact of miRNAs in HCC.^18^, ^19^ It is previously reported that miR‐1225 expression is repressed in human gastric carcinoma and inhibits metastasis and proliferation of malignant cells.^20^ Aberrant excessive miR‐1225 expression repressed migration, proliferation, and invasion of glioblastoma cells in vitro. Furthermore, excessive miR‐1225 expression impaired the growth of glioblastoma xenograft malignancy by targeting insulin receptor substrate 1 (IRS1).^21^ Additionally, miR‐1225 serves as a malignancy repressor in laryngeal and pancreatic malignant cells by targeting CDC14B and JAK1.^22^, ^23^

This study examines the impact of miR‐1225 on HCC cells. Repressed miR‐1225 expression was revealed in HCC tissue specimens and in HCC cells. Mechanistic exploration proved that miR‐1225 inhibited the migration, invasion, and proliferation of HCC cells in vitro. Furthermore, miR‐1225 was found to directly target NFκB p65 in HCC.

## 2 MATERIAL AND METHODS

### 2.1 Clinical samples

The study group included ten cases of HCC, with the age of the subjects ranging from 30‐60 years. Clinicopathological characteristics were acquired from medical records. We harvested the para‐neoplastic specimens at no less than 1 cm from the malignant specimens. All cases were hepatectomy candidates from Jining No.1 People's Hospital. The study was approved by the Ethical Committee of the Jining No.1 People's Hospital. Fully informed consent was acquired from each patient and doctor with regard to specimen usage. Diagnosis and review were performed independently by three pathologists.

### 2.2 Cells and transfection

Human Huh‐7 and HepG2 cells were acquired from the Cell Bank of Academia Sinica (Shanghai, China), and were cultured in DMEM medium with 10% FBS. Cells were maintained at 37°C with 5% CO_2_. Six‐well and 12‐well plates were utilized to seed 5 × 10^6^ cells per well, followed by overnight incubation until 80%‐90% confluency.

### 2.3 MiR‐1225 mimic/prohibitor preparation

The miR‐1225 mimic and negative control (NC) were obtained from RiboBio. NC and the miR‐1225 mimic were added to 0.9% NaCl to a final concentration of 10 mg/mL.

### 2.4 P65 overexpression

For p65 overexpression, p65 gene was inserted into the plasmid pCDNA3. The plasmid pCDNA3‐p65 was added to 50 µL of RNase free water at 100 nmol/µL. For transfection, 5 × 10^6^ HCC cells, Huh‐7 and HepG2, were seeded onto six‐well plates, followed by transfection with 2 µg of pCDNA3‐p65 or pCDNA3.

### 2.5 MTT Assessment

MTT assay was performed to assess cell proliferation. Briefly, cells were supplemented with twenty microliters of MTT (0.5 mg/mL) and incubated for four hours at 37°C. The supernatant was discarded, and 150 µL of DMSO was added to every well and rotated for ten minutes to dissolve the purple formazan crystals formed. Absorbance was measured on an Infinite M200 microplate reader (Tecan) at 490 nm.

### 2.6 EdU proliferation test

The cells were washed three times with PBS and then stained with 300 µL of EdU staining solution for 2 hours according to the manufacturer's protocol. After an additional three washes with PBS, the cells were examined with a fluorescence microscope (Olympus). Proliferating cells were identified by red staining, and the number of proliferating cells in six different fields was counted.

### 2.7 Western blotting

For protein extraction, cells were lysed in RIPA Lysis Buffer (Cell Signaling Inc). Equal amounts of total cellular protein were subjected to SDS‐PAGE and transferred to a PVDF membrane. The proteins were probed using the respective antibodies. The information for antibodies were showed as follows: MMP‐9 ab (1:1000, ab38898, Abcam), p65 ab (1:2500, ab16502, Abcam), and beta‐actin ab (1:5000, ab8227, Abcam).

### 2.8 RNA extraction and quantitative PCR

Total RNA was separated from cells using TRIzol reagent. For, qPCR, the reaction mixture included cDNA, forward and reverse primers, as well as SYBR Green PCR Master Mix in a total volume of 20 µL and was amplified in the Light‐Cycler 480 Real‐Time PCR system (Roche, Basel, Switzerland). GAPDH served as the internal reference. Quantification was carried out using the 2^−ΔΔ^ *^C^* ^T^ approach via normalization against GAPDH.

### 2.9 Transwell migration assay

HCC cells were trypsinized after 24 hours of transfection and washed with D‐Hanks solution. Matrigel inserts (eight micrometer pore size) were placed onto 24‐well plates to form the upper and lower compartments. Next, 400 µL of F‐12 medium with 10% FBS and 20 ng/mL HGF was added to the lower chamber whereas cells were seeded in the upper chamber. Migrated cells were stained with crystal violet and examined under a microscope (Zeiss).

### 2.10 Wound healing test

Cells (1 × 10^5^ cells/mL) were seeded in 24‐well plated after 48 h of treatment and were incubated until 70%‐80% confluency. Using a sterile pipette tip, a scratch wound was created by scraping the cell monolayer. Fresh medium was added, and the cells were incubated for further 48 hours or were not incubated (control). Cells were imaged under an inverted microscope (Nikon).

### 2.11 Dual‐luciferase reporter test

miR‐1225 was predicted to target the 3′‐UTR of p65. Thus, a wild type or mutated sequence of the predicted target site was generated. Dual‐Luciferase Reporter Assay system was use to examine luciferase function at 48 hours subsequent to transfection as instructed (Promega). Renilla luciferase function was used to normalize the firefly luciferase activity.

### 2.12 Statistical analysis

Outcomes were displayed as mean ± SD. One‐way ANOVA or Student's *t* tests were applied to evaluate the differences. *P* < .05 was considered significant.

## 3 RESULTS

### 3.1 MiR‐1225 expression is decreased in HCC cells and in tissue specimens from HCC patients

**Figure 1:**
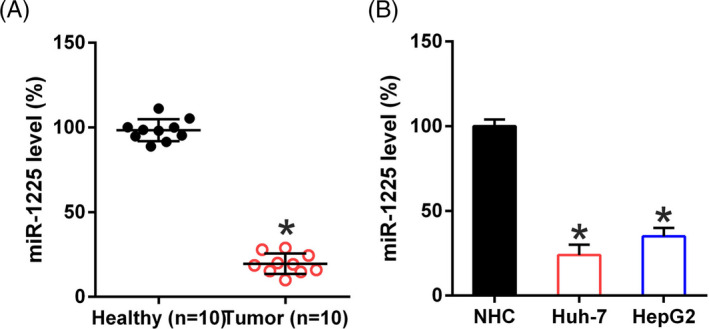
miR‐1225 expression in HCC cell lines and specimens. (A) miR‐1225 expression in specimens obtained from HCC patients (n = 10) vs normal healthy tissue (n = 10). (B) miR‐1225 levels in Huh‐7, HepG2, and normal hepatic cells were determined by qPCR. Outcome is displayed as mean ± SD. *P < .05 vs control group

Expression of microRNAs is relevant for the prognosis and diagnosis of different cancers. Our research revealed that miR‐1225 was remarkably downregulated in HCC samples compared to that in normal healthy samples (Figure 1A). miR‐1225 concentration in HepG2 and in Huh‐7 cells was compared with that in normal human hepatic cells (NHC) by qPCR. MiR‐1225 levels in HCC cells were lower than those in healthy cells (Figure 1B), indicating that miR‐1225 could contribute to properties of HCC cells.

### 3.2 MiR‐1225 repressed the proliferation of HCC cells

**Figure 2:**
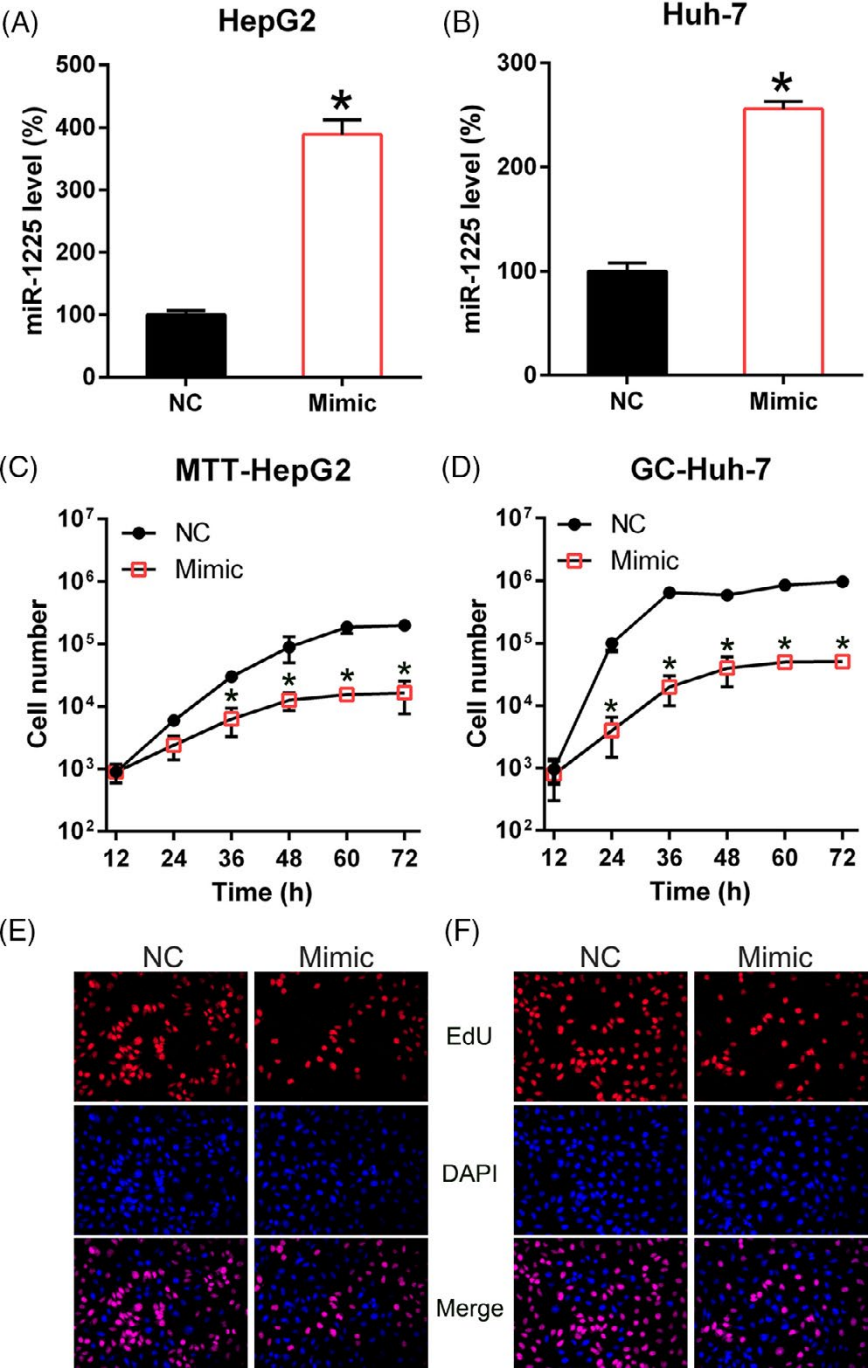
miR‐1225 upregulation suppresses the proliferation of Huh‐7 and HepG2 cells. The cell lines were transfected with the miR‐1225 mimic and NC mimic. qPCR (A, B) was conducted to confirm miR‐1225 upregulation in both cell lines. (C, D) Proliferation of Huh‐7 and HepG2 cells was measured at 12, 24, 36, 48, 60, and 72 hours after transfection using the MTT assay. (E, F) CCK‐8 assay of the Huh‐7 and HepG2 cells transfected with miR‐1225 mimic and NC mimic was observed by fluorescence microscope. Outcome is displayed as mean ± SD. *P < .05 vs control group

To confirm that miR‐1225 regulates survival and proliferation of HCC cells, Huh‐7 and HepG2 cells were transfected with a miR‐1225 mimic or NC mimic (Figure 2A, 2B). MTT assay showed that proliferation of Huh‐7 and HepG2 cells was reduced subsequent to transfection by the miR‐1225 mimic at 12‐72 hours post‐transfection (Figure 2C, 2D). miR‐1225 upregulation caused a noticeable decrease in CCK‐8‐positive cell numbers, but transfection with the NC mimic did not influence cell proliferation assessed by CCK‐8 (Figure 2E, 2F).

### 3.3 MiR‐1225 impaired migration and invasion of HCC cells

**Figure 3:**
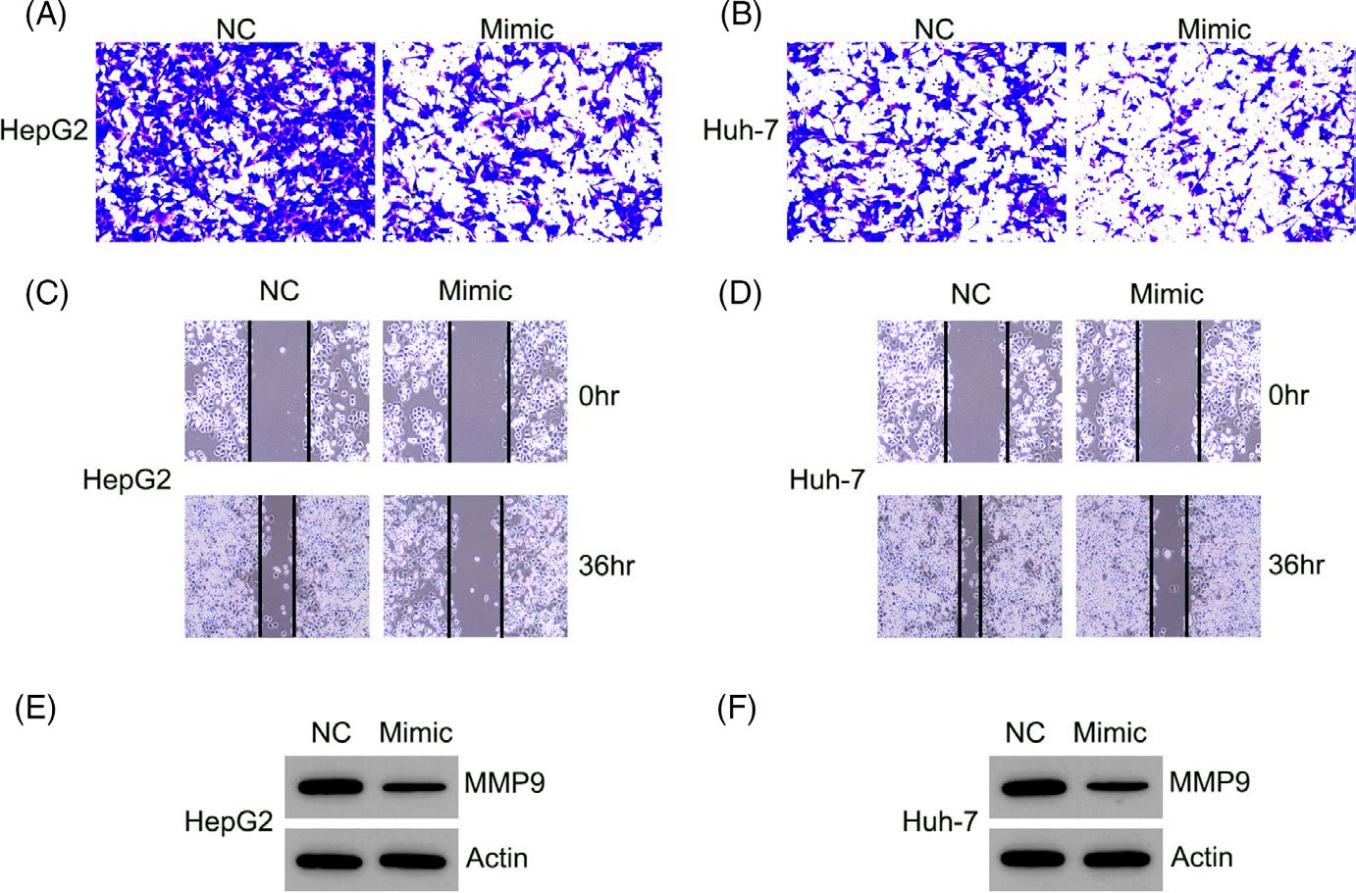
miR‐1225 upregulation inhibited HCC cell migration and invasion. Subsequent to transfection with the miR‐1225 mimic or NC mimic, (A, B) invasion and (C, D) migration of HepG2 and Huh‐7 cells was examined using the Transwell migration test and wound healing assay. (E, F) Western blotting was performed to determine the MMP‐9 expression in HCC cells expressing miR‐1225

Migration and invasion of HCC cells is a dominant contributor to mortality during HCC development and progression.^9^ To examine if miR‐1225 affects migration and invasion of HCC cells, Transwell migration and wound healing assays were performed subsequent to transfection of HepG2 and Huh‐7 cells with the miR‐1225 mimic. In the Transwell migration assay, miR‐1225 overexpression clearly decreased the invasion of HCC cells (Figure 3A, 3B). In the wound healing assay, miR‐1225 inhibited the migration of Huh‐7 and HepG2 cells toward the gap created by scratching the cell monolayer (Figure 3C, 3D), which was consistent with the outcome of the Transwell assay. Our study also explored the contribution of miR‐1225 to MMP‐9 expression, which is critical for migration and invasion capabilities of malignant cells. Ectopic upregulation of miR‐1225 led to a decrease in MMP‐9 in both cell lines, as determined by Western blotting (Figure 3E, 3F). These data suggest that miR‐1225 represses the invasive and migratory capacities of HCC cells in vitro.

### 3.4 MiR‐1225 targets the 3′‐UTR of NFκB p65

**Figure 4:**
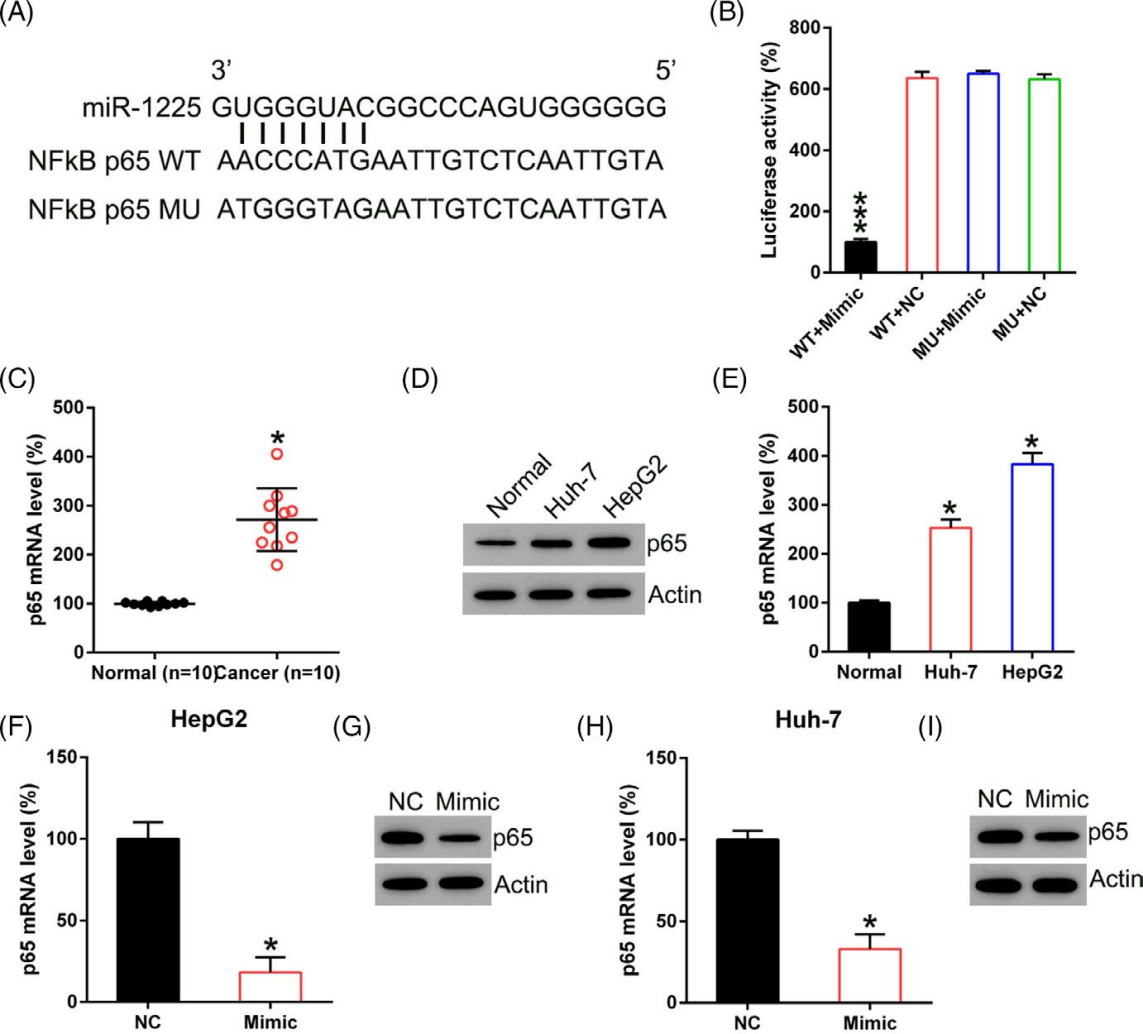
miR‐1225 targets p65. A, Graphical representation of the conserved miR‐1225 binding motif at the 3′‐UTR of NFκB p65. B, Luciferase function exhibited via luciferase reporter constructs carrying either the wild type (WT) or mutated (MU) human p65 3′‐UTR subsequent to miR‐1225 mimic transfection. Luciferase function was normalized to the function of Renilla luciferase. C, p65 expression in samples acquired from HCC patients (n = 10) vs. normal healthy tissue (n = 10) was examined by qPCR. D, Western blotting and E, qPCR were carried out in order to examine p65 expression in Huh‐7, HepG2, and normal liver cells. F, G, Western blotting and H, I, qPCR were conducted to assess p65 protein expression and transcription in HCC cells, respectively, subsequent to transfection with the miR‐1225 mimic and NC mimic. Outcome is displayed as mean ± SD. *P < .05, ***P < .001 vs control group

Bioinformatics analysis suggested that miR‐1225 could target the 3′‐UTR of NFκB p65 (Figure 4A), an essential regulator of human HCC.^24^ Dual‐Luciferase reporter assay was used to evaluate the direct association between the p65 3′‐UTR and miR‐1225 (Figure 4B). Luciferase activity was repressed by eighty‐five percent in cells that were transfected with the miR‐1225 mimic fused to the p65 3′‐UTR compared to the control group.

MiR‐1225 expression was subsequently assessed in HCC cells and in tissues. HCC displayed p65 upregulation compared to that in normal liver tissues (Figure 4C). NFκB p65 concentration in HepG2 and Huh‐7 cells was elevated compared with that in normal liver cells (Figure 4D, 4E). Western blotting and qPCR were utilized to assess the contribution of miR‐1225 mimic to p65 expression in HCC cells. Transcription and translation of p65 were reinforced subsequent to transfection with the miR‐1225 mimic (Figure 4F‐4I). It was thus proved that p65 was downregulated subsequent to miR‐1225 silencing and that miR‐1225 targeted the p65 3′‐UTR.

### 3.5 P65 overexpression counteracts the repressive impact of miR‐1225 on the proliferation, migration, and invasion of HCC cells

**Figure 5:**
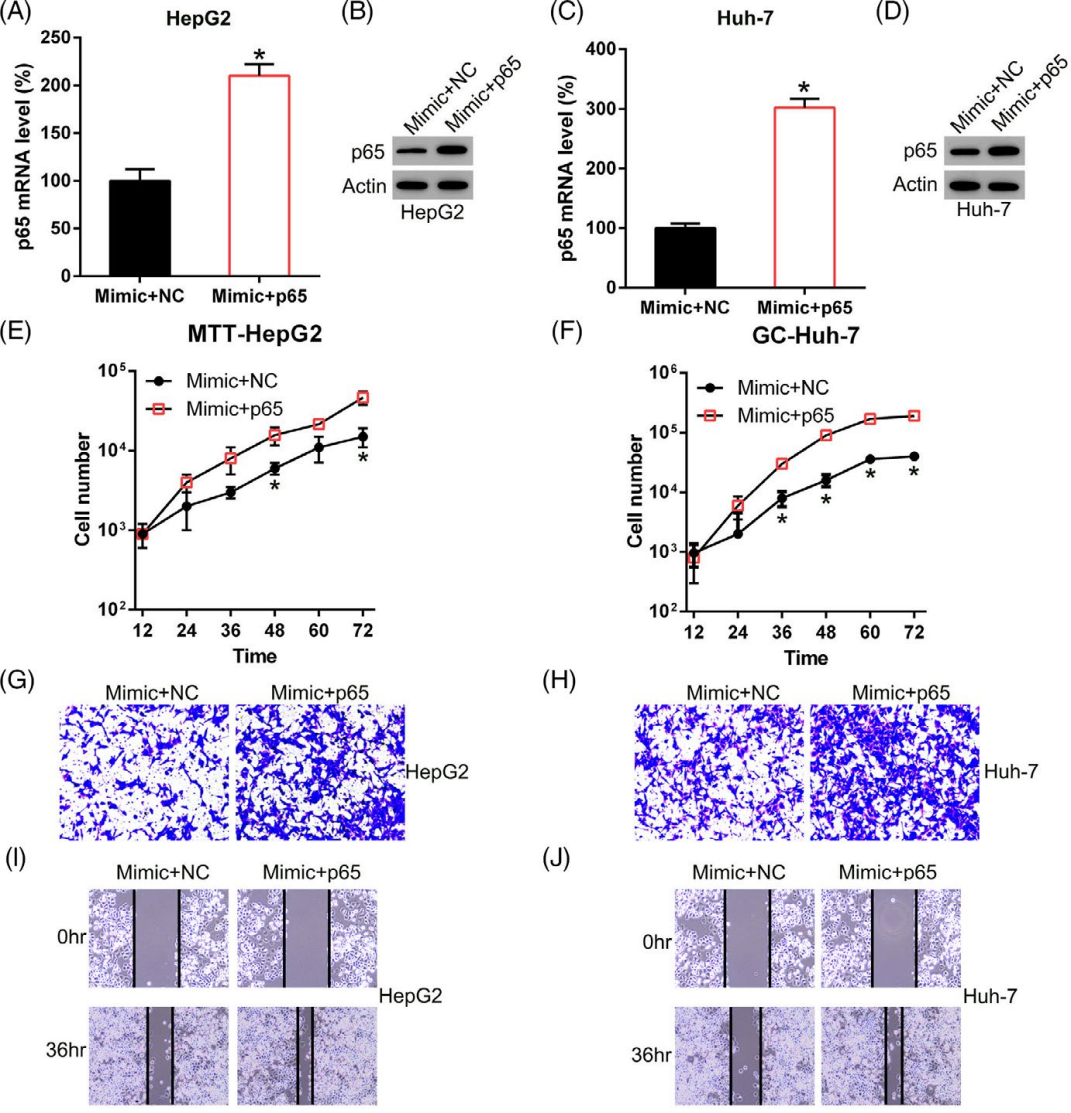
NFκB p65 is involved in miR‐1225‐inhibited proliferation, invasion, and migration of HepG2 and Huh‐7 cells. A, B, HepG2 and Huh‐7 cells underwent co‐transfection with of pCDNA3‐p65/pCDNA3 and the miR‐1225 mimic to upregulate the p65 expression. A, C, qPCR and B, D, WB were performed to detect the p65 protein expression in both HCC cell lines. E, F, Proliferation of Huh‐7 and HepG2 cells was measured at 12, 24, 36, 48, 60, and 72 hours after transfection, using the MTT assay. G, H, Invasion and I, J, migration of HepG2 and Huh‐7 cells was examined using the Transwell migration test and wound healing assay. Outcome is displayed as mean ± SD. *P < .05 vs control group

To assess whether p65 contributes to the repressive impact of miR‐1225 on the properties of HCC cells, p65 was overexpressed in two HCC cell lines, that also underwent transfection with the miR‐1225 mimic. Western blotting and qPCR were used to confirm p65 upregulation in both HCC cell lines (Figure 5A‐5D). p65 overexpression led to the proliferation recovery of those two cell lines repressed by miR‐1225, as evidenced by the MTT assay (Figure 5E, 5F). Upregulation of p65 resulted in a remarkable increase in the quantity of invasive HCC cells subsequent to transfection, using the Transwell assay (Figure 5G, 5H). Further, p65 remarkably increased the quantity of migrated Huh‐7 and HepG2 cells, as determined by the wound healing assay (Figure 5I, 5J). Thus, p65 shows a protective role on the migration and proliferation of HCC cells.

## 4 DISCUSSION

Mechanisms underlying HCC development are synergistic processes related to the transcription of various genes and multiple signal transductions. Although previous studies have explored HCC, an understanding of the molecular mechanisms underlying its development is currently poor. Many studies have revealed that miR‐1225 is relevant to the generation of various malignant tumor cells,^18^, ^19^, ^20^, ^21^, ^22^, ^23^ and however, its role in HCC is yet to be studied and clarified. In this study, miR‐1225 was found to be downregulated in tumor samples from HCC patients and in HCC cell lines. Further, miR‐1225 suppressed the expression of NFκB p65, a key modulator in various tumors. The miR‐1225 mimic repressed the HCC cell proliferation and was proved to directly bind to the p65 3′‐UTR, leading to p65 downregulation. Our results indicated that miR‐1225 exerts an oncogenic impact by targeting p65 and offers an innovative strategy to treat HCC.

Nuclear factor kappa B (NFκB) is a critical survival factor in various physiological reactions, such as embryonic liver development and liver regeneration.^25^ In liver cancers, NFκB is a dominant contributor to the resistance to TNF cytotoxicity and to functional pathways such as TNF receptor‐associated factor 2.^26^ NFκB serves as an essential regulator in the suppression of cell death reactions, and NFκB stimulation has been revealed to elevate cell death counteracting threshold of cells and tissues exposed to cytotoxic cytokines including TNF by repressing the triggering of caspase‐8 stimulation.^27^ On the contrary, NFκB exerts various wide‐ranging impacts regulated via a complicated modulating network of repressors and coactivators.^28^ Proteases like MMP‐9 are required for the invasion and destruction of extracellular matrix, thus boosting the invasive progression of cancerous cells to nearby healthy tissues.^29^, ^30^, ^31^ MMP‐9 from tumor and stromal cells, particularly macrophagocytes, has an indispensable position in the invasive, migratory, and angiogenic progression of malignant tumors. As NFκB is the key transcription factor for MMP9 expression,^32^ we hypothesize that NFκB could participate in regulating cancer cell metastasis by mediating MMP‐9 expression. The current study showed that downregulated miR‐1225 or increased NFκB expression in HCC samples was related to increased proliferation, migration, invasion, and enhanced MMP‐9 expression. Our results demonstrate that miR‐1225, to some extent, could downregulate NFκB‐induced MMP‐9 elevation in HepG2 and Huh‐7 cells.

In conclusion, this research indicates a malignancy counteracting impact of miR‐1225 on 2 HCC cell lines and suggest that HCC progression involves NFκB. However, we are still unable to rule out other potential factors that could be targeted by miR‐1225 and thus lead to HCC progression. Thus, additional cross talk assays are needed in future to explore and recognize the targets for biotin‐labeled miR‐1225 in HCC tissue specimens from patients and cell lines. This approach could offer a more accurate and profound interpretation for the contribution of miR‐1225 to HCC generation.
